# Activating Wnt/β-Catenin Signaling in Osteocytes Promotes Osteogenic Differentiation of BMSCs through BMP-7

**DOI:** 10.3390/ijms232416045

**Published:** 2022-12-16

**Authors:** Yining Zhang, Yixin Zhao, Zhengsong Xie, Molin Li, Yujiao Liu, Xiaolin Tu

**Affiliations:** Laboratory of Skeletal Development and Regeneration, Institute of Life Sciences, Chongqing Medical University, Chongqing 400016, China

**Keywords:** osteocytes, stem cells, bone formation, Wnt signaling, bone morphogenetic protein-7

## Abstract

Bone formation is critically needed in orthopedic clinical practice. We found that, bone morphogenetic protein-7 (BMP-7) gene expression was significantly increased in fractured mice, which activates canonical Wnt signaling exclusively in osteocytes. Wnt and BMP signaling appear to exhibit synergistic or antagonistic effects in different kinds of cells. However, the communication between Wnt/β-catenin signaling and BMP signaling in osteocytes is almost unknown. Our study verified in vitro that BMP-7 expression was significantly increased when Wnt signaling was activated in osteocytes. Next, BMP-7 in osteocytes was overexpressed using an adenovirus, the osteogenesis of bone marrow stem cells (BMSCs) was enhanced, when cocultured with osteocytes. On the contrary, BMP-7 in osteocytes was silenced using an adenovirus, the osteogenesis of bone marrow stem cells (BMSCs) was weakened. In addition, the osteogenesis of BMSCs was no longer promoted by Wnt-activated osteocytes when BMP-7 was silenced. Therefore, the results showed that BMP-7 mediated the anabolic actions of Wnt/β-catenin signaling in osteocytes. Our study provides new evidence for the clinical application of BMP-7-overexpressed osteocytes.

## 1. Introduction

Bone formation is critically needed in orthopedic clinical practice. The limited stability and differentiation of mesenchymal stromal cells make the osteogenic-inducing microenvironment necessary [1]. Osteocytes, the most abundant cells in bone tissue, integrate and transmit hormonal and mechanical signals that coordinate the functions of osteoblasts and osteoclasts. In addition, osteocytes affect cells in both the bone/bone marrow niche by paracrine mechanisms and distant organs by endocrine mechanisms [2]. Osteocytes activating canonical Wnt/β-catenin signaling provide an osteogenic microenvironment for BMSCs via the Notch signaling pathway [3]. The osteogenic effect of Wnt-activated osteocytes on BMSCs was also demonstrated in a three-dimensional environment [4]. On the contrary, sclerostin, an inhibitor of Wnt signaling, is secreted specifically by osteocytes and negatively regulates the osteogenic differentiation of BMSCs [5,6]. In addition, overexpressing Dll4 in osteocytes promotes the osteogenic differentiation of BMSCs via the RBPjκ-dependent canonical Notch signaling pathway [7]. We found the BMP-7 expression increased in Wnt-activated osteocytes. However, until now, the relation of BMP-7 and Wnt signal in osteocytes in bone formation is almost unknown.

The synergistic or antagonistic effects of Wnt and BMP signaling may depend on the target cell type or the different stages of target cells. BMPs are recognized as potent bone induction factors, and BMP-2, BMP-4, BMP-6, BMP-7, and BMP-9 promote osteogenic differentiation of BMSCs in vitro and in vivo [8,9,10,11]. β-catenin synergistically promotes osteoblast differentiation with BMP-2, and BMP-2 mediated by Wnt signaling increases ALP expression and mineralization in preosteoblasts [12,13]. Conversely, BMP-3, a negative regulator of BMP signaling, has been proven to be downstream of the Wnt signaling [14]. In addition, BMP signaling in osteocytes antagonizes the Wnt signal by inducing sclerostin secretion. Sclerostin is reduced after the knockout of BMPR1 in osteocytes, weakening its inhibition of Wnt [15]. In contrast, BMPR1 deletion significantly inhibits Wnt genes in cortical bone [16]. Runx1 has been confirmed in recent studies to regulate bone homeostasis by increasing BMP-7/Alk3/Smad1/5/8/Runx2/ATF4 and Wnt signaling. BMP-7 overexpression can save Alk3, Runx2, and ATF4 expression in Runx1-deleted BMSCs, indicating that BMP and Wnt signaling can be coordinated by Runx1 [17]. Activation of Wnt signaling in mice osteocytes (daβcat^ot^ mice) promotes bone anabolism and bone formation [18], but the link between Wnt signaling and BMP signaling in osteocytes remains unclear.

Our study aimed to investigate the role of BMP-7 in Wnt/β-catenin-activated osteocytes in regulating osteogenic differentiation. We hypothesized that BMP-7 mediates the anabolic actions of Wnt/β-catenin signaling in osteocytes. To confirm our hypothesis, we first measured the expression of BMP-7 in osteocytes activated by Wnt/β-catenin in vivo and in vitro. Subsequently, the osteogenic differentiation of BMSCs was examined when cocultured with the osteocytes overexpressed or silenced BMP-7. Finally, BMP-7-silenced osteocytes were treated with Wnt3a to investigate the osteogenic differentiation in the coculture system. These specific cellular responses could elucidate the role of BMP-7 in Wnt/ β-catenin-mediated bone anabolism in osteocytes. These results would become a critical step in translational application.

## 2. Results

### 2.1. BMP-7 Is Highly Expressed in Osteocytes of daβcat^ot^ Mice Which Activating Wnt/β-Catenin Signaling in Osteocytes

**Figure 1:**
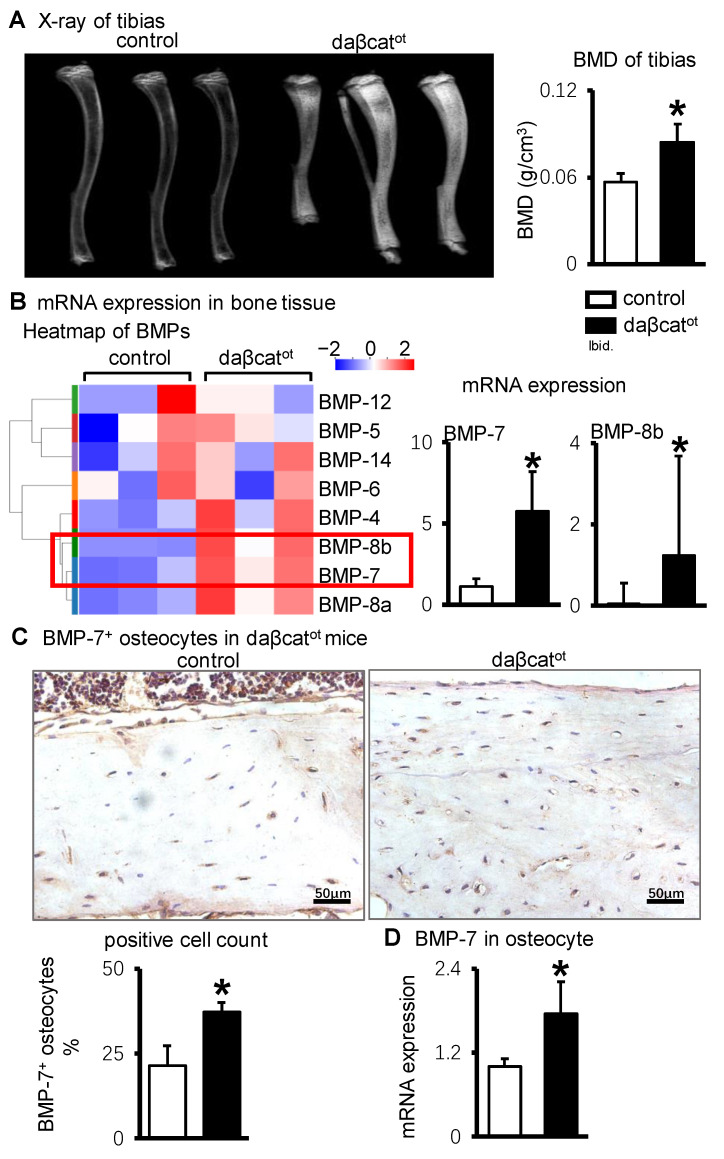
BMP-7 expression in bone tissue and osteocytes of daβcat^ot^ mice. (**A**). X-ray images and BMD quantification results. (**B**). Cluster analysis heat map of BMP signal in fractured tibial bone tissue of daβcat^ot^ mice and differential gene expression of BMP-7, two significantly increased genes are in the red box. (**C**). Immunohistochemical staining of BMP-7 in osteocytes and quantitative analysis of BMP-7-positive osteocytes in tibial cortical bone of daβcat^ot^ mice. (**D**). RT-qPCR analysis of BMP-7 gene expression in primary osteocytes of daβcat^ot^ mice. * *p* < 0.05, compared with control group.

The daβcat^ot^ mice aged 8–10 weeks were used for in vivo experiments and cre^-^ mice from the same litter were considered controls. The X-ray (Figure 1A) showed that daβcat^ot^ mice exhibited higher bone mineral density than the control group. BMD quantification results (Figure 1A) were consistent with X-ray images. Tissue RNA from fractured mice tibias was collected for gene sequencing. Cluster analysis (Figure 1B) showed BMP signaling increased in the tibias of daβcat^ot^ mice compared with control mice. BMP-7, BMP-8b were significantly increased in daβcat^ot^ mice. BMP-7 immunohistochemistry showed osteocytes were abundant in the tibial cortical bone of daβcat^ot^ mice compared with control mice, and the proportion of BMP-7-positive osteocytes was significantly increased in daβcat^ot^ mice (Figure 1C). Subsequently, primary osteocytes were isolated from daβcat^ot^ mice. The qPCR results showed BMP-7 gene expression was significantly increased in osteocytes of daβcat^ot^ mice compared with control mice (Figure 1D).

### 2.2. In Vitro: BMP-7 Is Highly Expressed in Osteocytes When Osteocytes Activate Wnt/β-Catenin Signaling

**Figure 2:**
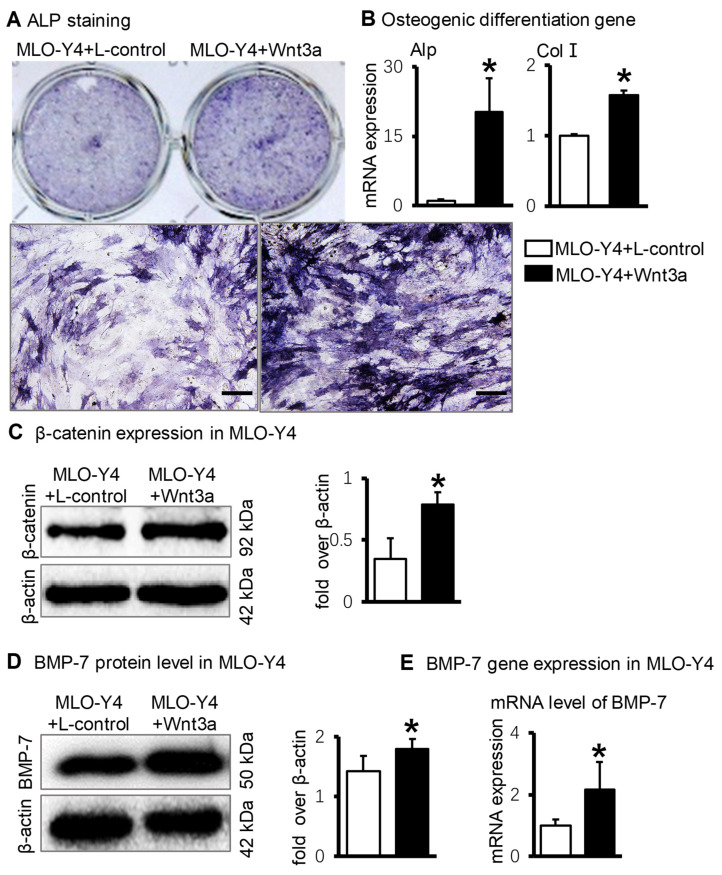
BMP-7 expression in Wnt-activated osteocytes in vitro. (**A**). ALP staining in the coculture system of Wnt3a-treated MLO-Y4 cells and ST2 cells. (**B**). Osteogenic marker gene expression in the coculture system of Wnt3a-treated MLO-Y4 cells and ST2 cells. (**C**). The protein level of β-catenin in MLO-Y4 cells after treatment with Wnt3a by Western blotting. (**D**). The protein level of BMP-7 in MLO-Y4 cells after treatment with Wnt3a based on Western blotting. (**E**). The gene expression of BMP-7 in MLO-Y4 cells after treatment with Wnt3a using RT-qPCR. * *p* < 0.05, compared with MLO-Y4+L-control group. Scale bar: (**A**) 50 μm.

For in vitro experiments, the MLO-Y4 cells were stimulated with Wnt3a supernatant for 48 h and then cocultured with ST2 cells in a 1:4 ratio for 3 days. ALP staining results showed that Wnt3a-activated MLO-Y4 cells exerted a more potent effect on promoting osteogenic differentiation of ST2 cells (Figure 2A). The regulatory protein of Wnt signaling, β-catenin, was increased in MLO-Y4 after treatment with Wnt-3a (Figure 2C). Osteogenic marker genes Alp and Col Ⅰ were significantly increased in the coculture system (Figure 2B). Simultaneously, the BMP-7 protein level (Figure 2D) and gene expression (Figure 2E) in Wnt3a-activated MLO-Y4 cells were significantly increased.

### 2.3. BMP-7-Overexpressed Osteocytes Promote Osteogenic Differentiation of ST2 Cells

Whether highly expressed BMP-7 in osteocytes induced by the Wnt signal is beneficial to osteogenic differentiation. First, the effects of osteocytes that highly expressed BMP-7 on the osteogenic differentiation of ST2 cells were investigated. 

**Figure 3:**
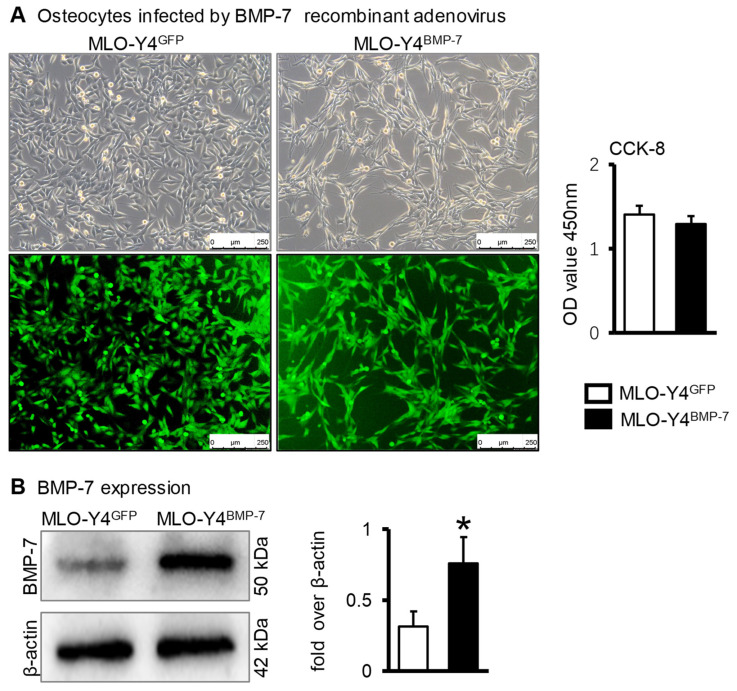
The construction of osteocytes with high expression of BMP-7. (**A**). Fluorescence image and proliferation of MLO-Y4 cells after transfection of Ad-BMP-7. (**B**). Western blotting validated the BMP-7 protein level in MLO-Y4 cells transfected with Ad-BMP-7. * *p* < 0.05, compared with MLO-Y4^GFP^ group.

A recombinant adenovirus overexpressing BMP-7 (Ad-BMP-7) was constructed. Green fluorescence was observed in the MLO-Y4 cells when Ad-BMP-7 or Ad-GFP (control group) was effectively infected. In this case, 80% of the cells in each group were successfully infected after 48 h. CCK-8 results showed that there was no significant difference in cell proliferation between Ad-BMP-7 and Ad-GFP group (Figure 3A); Western blotting analysis showed BMP-7 was highly expressed in MLO-Y4 cells infected with Ad-BMP-7 compared with Ad-GFP (Figure 3B), indicating a successful transfection. Therefore, the transfected cells were suitable for further experimental use.

**Figure 4:**
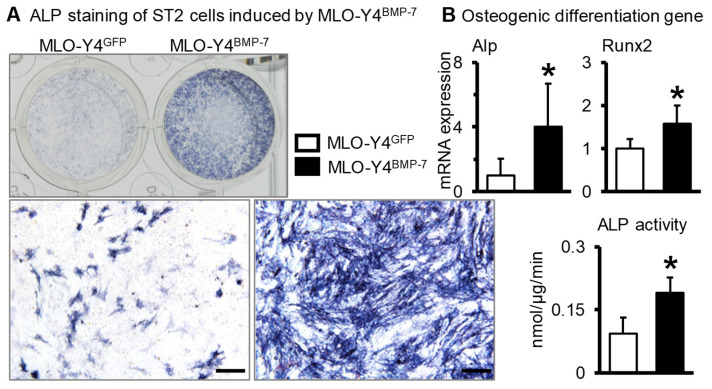
The osteogenic differentiation of ST2 cells when cocultured with BMP-7-overexpressed osteocytes. (**A**). ALP staining and ALP activity in the coculture system of ST2 cells and BMP-7-overexpressed MLO-Y4 cells. (**B**). Osteogenic gene expression in the coculture system of ST2 cells and BMP-7-overexpressed MLO-Y4 cells. * *p* < 0.05, compared with MLO-Y4^GFP^ group. Scale bar: 50 μm.

BMP-7/GFP-overexpressed MLO-Y4 cells were cocultured with ST2 cells in a 1:4 ratio. ALP staining and quantitative ALP activity (Figure 4A) showed osteogenesis was more evident after 3 days of coculture in ST2+MLO-Y4^BMP−7^ cells compared with ST2+MLO-Y4^GFP^ cells. Osteogenic marker genes of the cocultured cells were detected using qPCR (Figure 4B), while Alp and Runx2 gene expression in ST2+MLO-Y4^BMP−7^ cells was significantly higher than in ST2+MLO-Y4^GFP^ cells.

### 2.4. MLO-Y4 Cells Silencing BMP-7 Inhibited the Osteogenic Differentiation of ST2 Cells

**Figure 5:**
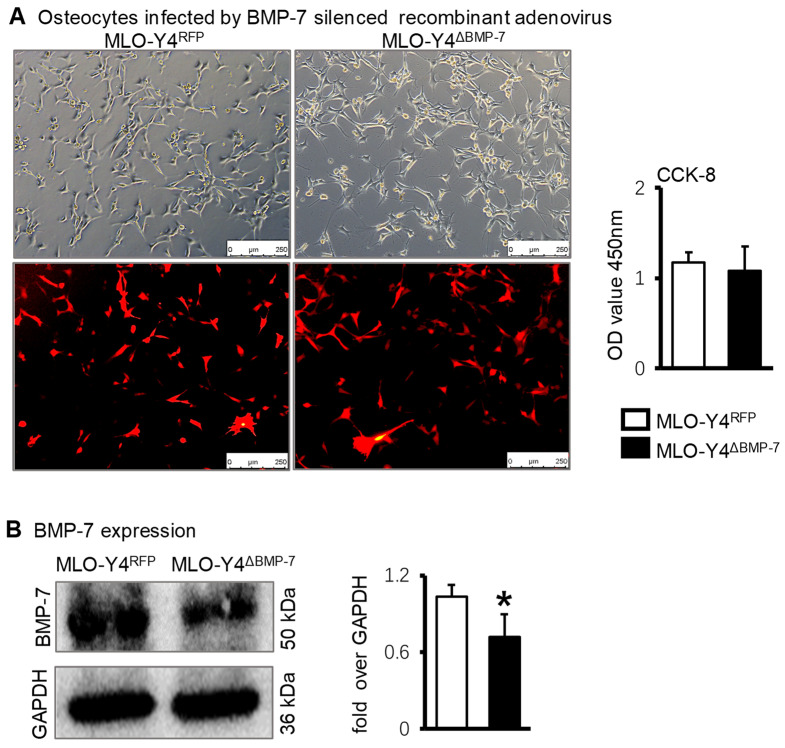
The construction of osteocytes silencing BMP-7. (**A**). Fluorescence image and proliferation of MLO-Y4 after transfection of Ad-siBMP-7. (**B**). BMP-7 protein expression in MLO-Y4 cells after silencing BMP-7 based on Western blotting results. * *p* < 0.05, compared with MLO-Y4^RFP^ group.

Next, the effects of lowly expressed BMP-7 osteocytes on osteogenic differentiation were investigated. An adenovirus silencing BMP-7 (Ad-siBMP-7) was constructed. Red fluorescence was observed when MLO-Y4 cells were successfully transfected with Ad-RFP (control group) and Ad-siBMP-7. The transfection rate was approximately 80% after 48 h. CCK-8 results showed that there was no significant difference in cell proliferation between Ad-siBMP-7 and Ad-RFP group (Figure 5A). The BMP-7 protein level in MLO-Y4 cells was measured after successful transfection and Western blotting results showed Ad-siBMP-7 effectively inhibited BMP-7 expression in MLO-Y4 cells (Figure 5B).

**Figure 6:**
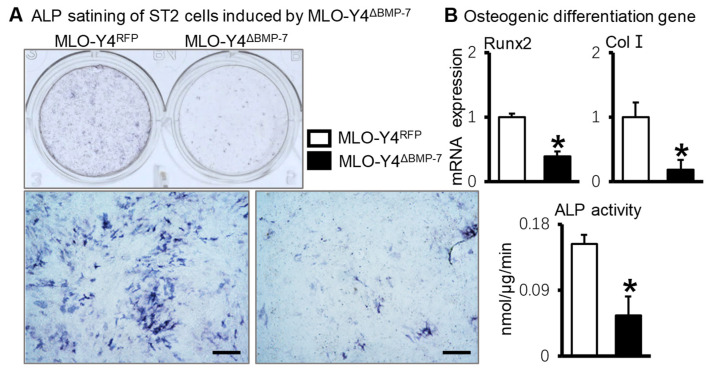
The osteogenic differentiation of ST2 cells when cocultured with BMP-7-silenced MLO-Y4 cells. (**A**). ALP staining and quantitative analysis of ALP activity in the coculture system of BMP-7-silenced MLO-Y4 cells and ST2 cells. (**B**). The osteogenic gene expression in the coculture system of BMP-7-silenced MLO-Y4 cells and ST2 cells. * *p* < 0.05, compared with MLO-Y4^RFP^ group. Scale bar: 50 μm.

Subsequently, BMP-7-silenced MLO-Y4 cells were cocultured with ST2 cells in a 1:4 ratio for 3 days. Compared with ST2+MLO-Y4^RFP^ cells, ALP staining and ALP activity (Figure 6A), and gene expression of osteogenic markers were significantly decreased in ST2+MLO-Y4^ΔBMP−7^ cells (Figure 6B). These results showed MLO-Y4 cells silencing BMP-7 inhibited the osteogenic differentiation of ST2 cells in the MLO-Y4 environment.

### 2.5. BMP-7-Silenced MLO-Y4 Cells Cannot Promote Osteogenic Differentiation of ST2 Cells, Even Though Wnt3a Stimulates These MLO-Y4 Cells

**Figure 7:**
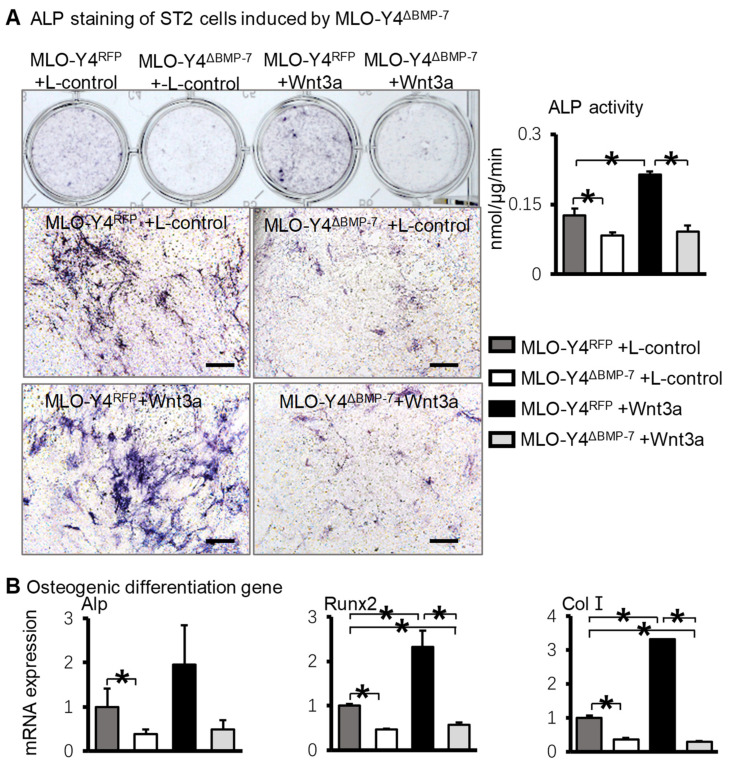
The osteogenic differentiation of ST2 cells when cocultured with BMP-7-silenced MLO-Y4 cells, which were pretreated Wnt3a. (**A**). ALP staining and quantitative analysis of ALP activity in the coculture of ST2 cells and BMP-7-silenced MLO-Y4 cells treated with Wnt3a. (**B**). The osteogenic gene changes in the coculture of ST2 cells and BMP-7-silenced MLO-Y4 cells treated with Wnt3a based on RT-qPCR. * *p* < 0.05, compared with the specified group. Scale bar: 50 μm.

Whether BMP-7 mediates the osteogenesis induced by the Wnt signal in osteocytes was investigated. MLO-Y4^RFP^ cells and MLO-Y4^ΔBMP−7^ cells were treated with Wnt3a or L-control (control cells) supernatant for 48 h, and then cocultured with ST2 cells in a 1:4 ratio for 3 days. ALP staining and ALP activity quantification (Figure 7A) showed the osteogenic differentiation of ST2 cells was significantly increased in the MLO-Y4^RFP^ environment when MLO-Y4^RFP^ cells were treated with Wnt3a supernatant compared with L-control supernatant. In contrast, compared with the MLO-Y4^RFP^ environment, the osteogenesis of ST2 cells in the MLO-Y4^ΔBMP−7^ environment was no longer increased when MLO-Y4^ΔBMP−7^ cells were treated with Wnt3a supernatant. The gene expression of osteogenic differentiation markers (Figure 7B) confirmed the results. These results indicated that BMP-7 mediates the osteogenic differentiation of ST2 cells in the osteocytic Wnt environment.

## 3. Discussion

The crosstalk between Wnt signaling and BMP-7 signaling in osteocytes is almost unknown. In our study, we sequenced the fracture tissue of daβcat^ot^ mice (mice activated Wnt/β-catenin signaling in osteocytes), activated Wnt signaling in osteocytes in vitro, and found BMP-7 expression was significantly increased in osteocytes. We further transfected osteocytes with adenovirus in order to overexpress BMP-7 and found the increased osteogenesis of BMSCs when coculture with BMP-7-activated osteocytes. On the contrary, when BMP-7 was silenced in osteocytes by adenovirus, the osteogenesis of BMSCs was weakened after coculture with BMP-7-silenced osteocytes. Finally, BMP-7-silenced osteocytes were treated with Wnt3a, and the osteogenesis of BMSCs in the coculture system was weakened.

Osteocytes are involved in fracture healing. In the early stage of fracture, osteocytes around the fracture site highly express inflammatory markers such as interleukin-6 (IL-6) and cyclooxygenase-2 (COX-2). Both the upregulation of osteocytic-specific markers such as E11 and DMP-1 and the downregulation of sclerostin promote osteogenesis. In the intermediate stage of fracture healing, osteocytes express growth factor BMP and cysteine-rich 61(CYR61), which can promote chondrogenesis [19]. Thus, osteocytes can provide a microenvironment for stem cell development. Therefore, we collected the fracture tissue of daβcat^ot^ mice one week after fracture for sequencing analysis. BMP-7 and BMP-8b increased, indicating that the above genes were closely related to Wnt signaling in osteocytes.

Multiple cells, such as osteocytes, osteoblasts, osteoclasts, and chondrocytes, exist in the tissue taken from the fracture site. Therefore, the osteocytic BMP-7 level could not be specifically explained based on gene sequencing results. In order to determine the BMP-7 level in Wnt-activated osteocytes, immunohistochemical staining of BMP-7 was performed on paraffin sections of the tibia of daβcat^ot^ mice, and BMP-7-positive osteocytes were counted. In addition, osteocytes were extracted from daβcat^ot^ mice and BMP-7 expression was detected and verified in vitro.

The applications of BMPs have been reported in nonunion, open fracture, aseptic osteonecrosis, and severe bone defects [20,21]. However, to obtain the desired bone-inducing effect, these proteins are always at super-physiological concentrations when applied directly and lead to safety problems, including explosive release and uncontrolled concentration [22,23]. Therefore, a carrier that can provide sustained, controlled, and effective concentration at the bone generation site is urgently needed [24].

Researchers attempted to achieve osteogenic effects around osteoblasts. However, activating the classical Wnt signaling of osteoblasts leads to leukemia [25]. When knocking out BMP type Ⅰ receptor A (BMPR1A) in osteoblasts and osteocytes, sclerostin decreased, and Wnt signaling increased, promoting bone mass and mechanical strength in mice [26,27]. However, in osteocytes, few studies discussed the relation between BMP signaling and Wnt signaling. Therefore, it needs further exploration.

BMP-7 was approved by the FDA for clinical use in spinal fusion in 2001. Although high costs and safety concerns hampered BMP-7, there is little debate regarding its treatment potential [28]. In order to accelerate the product’s clinical transformation, we studied BMP-7 first. Compared with the BMP-7 protein, we used osteocyte overexpressing BMP-7 and its supernatant as an osteogenic microenvironment. We suspected osteocytes would activate the specific pathway by BMP-7 and secrete other osteogenic factors besides BMP-7. We are planning to sequence the osteocytes overexpressing BMP-7 and its exosomes. We are working on the osteogenesis of osteocytes overexpressing BMP-8b. The bone-fat balance was decided by the lineage allocation of mesenchymal stem cells to adipocytes or osteoblasts [29]. Different from BMP-7, BMP-8b has been extensively studied in fat metabolism. For example, BMP-8b increases brown adipose tissue thermogenesis [30]. Based on the negative effect of BMP-8b on adipogenesis, it is reasonable to believe that the overexpression of BMP-8b in osteocytes promotes osteogenic differentiation.

In order to improve transfection efficiency, we selected adenovirus to transfect MLO-Y4 cells. Adenovirus cannot be integrated into the genome, and MLO-Y4 cells cannot express the target gene stably after passage [31]. In order to maintain the overexpression or silencing of BMP-7 in osteocytes, we needed to add adenovirus multiple times. However, MLO-Y4 cells and ST2 cells had been cocultured. The additional adenovirus would transfect into both MLO-Y4 cells and ST2 cells. Therefore, we need to plate enough transfected MLO-Y4 cells at the beginning. It made the coculture cells fill the plate in 3 days. Based on the above, we analyzed the bone formation after 3 days of coculture. The ultimate goal of our study is to build an osteogenic microenvironment to treat bone defects. In order to achieve mineralization, the experiment must involve a late coculture result of 5, 7, 14, 28 days. The stable expression of BMP-7 in MLO-Y4 cells can reduce the initial amount of MLO-Y4 cells in a coculture system. Lentiviruses may be a better choice.

In conclusion, this study showed that, in osteocytes, Wnt signaling promotes bone formation through overexpression of BMP-7. We aim to implant this BMP-7-activated osteocytic microenvironment in an injectable hydrogel to accelerate bone formation. We expect to provide evidence for the clinical transformation of the osteogenic microenvironment of BMP-7-activated osteocytes.

## 4. Materials and Methods

### 4.1. Animals

The control group and the daβcat^ot^ group, with 6 mice in each group (3 females and 3 males, respectively). In this case, daβcat^ot^ mice at 8–10 weeks of age are genetically modified mice used by our research group. The control mice came from the same litter of daβcat^ot^ mice [18]. Catnb lox (ex3) mice produce the daβcat^ot^ mice with dentin matrix protein 1 (DMP1)-8kb-Cre mice. The loxP sequence is inserted in the third exon of the Catnb gene, the β-catenin continuous phosphorylation site. The site is recognized by DMP1-8kb-Cre recombinase and spliced out to ensure β-catenin is stable and abundant in the cytoplasm because it is not degraded by phosphorylation. DMP1-8kb-cre is a recombinant enzyme that specifically expresses Cre only in osteocytes.

The mice were fed and operated on in the experiment center of Chongqing Medical University. The animals were kept in a conditioned room (24 ± 1 °C) with 12-h light/12-h dark cycles and free access to water and food. All animal experiments were performed in compliance with the institutional ethical requirements and approved by the Committee of Chongqing Medical University for the Use and Care of Animals.

### 4.2. Tissue Collection

In this case, daβcat^ot^ mice at 8–10 weeks of age were sacrificed and had their tibias collected. The tibias were fixed in 10% formalin overnight at room temperature on a shaker. Next, excess muscle was removed from the bone surface and bones immersed in 14% EDTA solution (pH 7.0), stirred continuously with a magnetic mixer in a 4 °C refrigerator for decalcification for 21 days.

### 4.3. Collection of Primary Osteocytes

The osteocytes were isolated from the tibia and femur as previously described [32]. The tibias and femurs were cut into pieces 1–2 mm in size and placed into 6-well plates. First, 1 mL type I collagenase (Sigma, St Louis, MO, USA) was added and the 6-well plates were placed in an 80 rpm shaker at 37 °C for 15 min. Type I collagenase was then removed and 1 mL complete medium added and the plates were kept on ice for 5 min. The first step was repeated twice. Second, the complete medium was removed and 1 mL of 4 mM EDTA solution added. The 6-well plates were placed in an 80 rpm shaker at 37 °C for 5 min. Next, EDTA was removed and 1 mL type Ⅰ collagenase added. The plates were placed in an 80 rpm shaker at 37 °C for 5 min. The second step was repeated twice. After treatment, the bone fragments were cultured at 37 °C in a 5% CO_2_ incubator.

### 4.4. Cell Culture

The osteocytic cell line MLO-Y4 cells and bone marrow stromal cell line ST2 cells were a gift from Professor Lynda Bonewald. The primary osteocytes were extracted from daβcat^ot^ mice. The MLO-Y4 cells, ST2 cells, and primary osteocytes were cultured in α-MEM complete medium (Thermo, Waltham, MA, USA) containing 10% fetal bovine serum (BI, Kibbutz Beit Haemek, Israel) and incubated at 37 °C in 5% CO_2_.

### 4.5. Transfection of Adenovirus in MLO-Y4 Cells

Ad-BMP-7 and Ad-siBMP-7 adenovirus were reconstituted using AdEasy plasmid system. The target fragment was first amplified by PCR and cloned into shuttle plasmids with high-fidelity Taq enzyme. The recombinant shuttle plasmid and adenovirus genome plasmid were co-transfected into 293 cells. BMP-7 overexpression and silencing recombinant adenovirus were obtained by labeling with green and red fluorescent proteins, respectively. Blank controls (Ad-GFP and Ad-RFP) were constructed in the same way.

MLO-Y4 cells were inoculated into 6-well plates at a density of 5 × 10^4^/well. After cell adherence, adenovirus Ad-BMP-7 (control group Ad-GFP) or Ad-siBMP-7 (control group Ad-RFP) was added, as well as polybrene (Sigma Aldrich, St. Louis, MO, USA) in a 1:1 ratio to the virus. After 48 h, the transfection effect was determined using a fluorescence microscope. Ad-BMP-7 and Ad-GFP were successfully transfected and showed green fluorescence, which were named MLO-Y4^BMP-7^ and MLO-Y4^GFP^ cells, respectively, in this research. Ad-siBMP-7 and Ad-RFP were successfully transfected and showed red fluorescence, which were named MLO-Y4^ΔBMP-7^ and MLO-Y4^RFP^ cells, respectively. The cells were collected after the transfection rate reached 70–80% and BMP-7 protein was used to verify transfection. The transfected MLO-Y4 cells were used for coculture with ST2 cells.

### 4.6. CCK-8 Assays

The MLO-Y4 transfected with Ad-GFP, Ad-BMP-7, Ad-RFP, and Ad-siBMP-7 was cultured into 96-well plates at 5000/well. After 24 h, 10 μL CCK-8 reagent (GLPBIO, Montclair, CA, USA) was added. After 4 h of culture, the optical density (OD) value at the wavelength of 450 nm was detected.

### 4.7. Coculture Assays

The 2 × 10^4^/well adenovirus-transfected MLO-Y4 cells and 8 × 10^4^/well ST2 cells were mixed and cocultured in 24-well plates for 3 days [4,7]. ST2 cells cocultured with BMP-7-overexpressed MLO-Y4 cells were named ST2+ MLO-Y4^BMP−7^ (control group ST2+ MLO-Y4^GFP^). ST2 cells cocultured with BMP-7-silenced-MLO-Y4 cells were named ST2+ MLO-Y4^ΔBMP−7^ (control group ST2+ MLO-Y4^RFP^).

### 4.8. Immunohistochemistry

Immunostaining was performed using a standard protocol. The sections were deparaffinized, subjected to 0.25% trypsin at 37 °C for 30 min to retrieve the antigen, treated with 3% H_2_O_2_ to inhibit endogenous peroxidase activity at room temperature for 20 min, blocked with 10% goat serum at room temperature for 30 min, and then incubated with rabbit polyclonal anti-BMP-7 antibody (1:100; Wanlei, Shenyang, China) overnight. On the second day, the sections were incubated with goat anti-rabbit secondary antibody (1:100, Bioss, Beijing, China). The color was developed using a diaminobenzidine substrate chromogen system (Bioss, Beijing, China). The cells expressing BMP-7 were stained brown and negative cells purple. Corresponding nonimmune IgGs were used as negative controls.

### 4.9. Alkaline Phosphatase (ALP) Assay

After 3 days of coculture, the cells in 24-well plates were rinsed with PBS and fixed with 4% neutral formaldehyde at room temperature for 10 min. The ALP staining reagent (Beyotime Biotechnology, Shanghai, China) was prepared according to the manufacturer’s instructions and 250 μL was added to each well followed by incubation in a dark room at room temperature until color developed. We used ALP kits (Beyotime Biotechnology, Shanghai, China) to quantitate ALP, cells in 24-well plates were cocultured for 3 days, then rinsed with PBS, and 300 μL Tris (50 mmol/L, pH = 7.4) was added to each well. The cells were collected into an Eppendorf tube and crushed using ultrasound and cooling. The cells were centrifuged at 12,000 rpm for 3 min at room temperature, the supernatant removed, and the protein concentration determined using the BCA assay (Beyotime Biotechnology, Shanghai, China). The standard sample and substrate were added following the manufacturer’s instructions, incubated at 37 °C, and the timing was started. When the working solution turned yellow, the termination solution was added and the timing stopped. The absorbance was measured at 405 nm with a microplate reader.

### 4.10. qPCR

Total RNA was extracted from the tibia using TRIzol (Invitrogen, Carlsbad, CA, USA). cDNA was synthesized by PrimeScript™ RT reagent kit with gDNA Eraser (Takara, Shiga, Japan) and used as the template for qPCR with primer sets (Table 1). The gene expression was analyzed by qPCR using SYBR^®^ Premix Ex Taq™ II (Takara, Shiga, Japan), and primer probe sets from Takara Bio Inc. Relative mRNA expression levels were normalized to the control group using the ^ΔΔ^Ct method. GAPDH or β-actin was used as the housekeeping gene.

### 4.11. Western Blotting

Protein lysates were prepared from the cells. The supernatant was extracted to determine the protein concentration using the BCA protein assay kit (Beyotime Biotechnology, Shanghai, China). Equal amounts of protein (30 μg) were run on a 10% SDS-polyacrylamide electrophoresis gel and transferred to a 0.22 PVDF membrane. The membranes were blocked for 2 h in 5% bovine serum albumin (BSA) in a shaker at 37 °C, then incubated with primary BMP-7 antibody (1:500; Wanlei, Shenyang, China) overnight at 4 °C, followed by incubation with anti-goat rabbit secondary antibodies (1:5000; Wanlei, Shenyang, China) in a shaker for 2 h at room temperature. The proteins were detected using an enhanced chemiluminescence (ECL) detection kit. The bands were finally quantified using ImageJ software (https://imagej.nih.gov (accessed on 26 June 2019)).

### 4.12. Statistical Analysis

The data were analyzed using GraphPad Prism 8 (https://www.graphpad.com/ (accessed on 12 March 2021)). All the results are presented with mean and standard deviation (SD). The differences between two and multiple groups were compared using two-tailed Student’s *t*-test and one-way analysis of variance (ANOVA). All results were repeated at least three times, and *p* ≤ 0.05 was considered to indicate statistical significance.

## 5. Conclusions

BMP-7 mediated the anabolic actions of Wnt/β-catenin signaling in osteocytes. The overexpression of BMP-7 in osteocytes promotes the osteogenesis of BMSCs.

## Figures and Tables

**Table 1:** The sequences of primers used for RT-PCR (mouse).

Primer	Forward	Reverse
Gapdh	GCACAGTCAAGGCCGAGAAT	GCCTTCTCCATGGTGGTGAA
beta-actin	AGAGGGAAATCGTGCGTGAC	CCATACCCAAGAAGGAAGGCT
Bmp-7	TCCAAGACGCCAAAGAACCAAGAG	CCTTCAGGTGCAATGATCCAGTCC
Alp	CACGGCGTCCATGAGCAGAAC	CAGGCACAGTGGTCAAGGTTGG
Col1	GACAGGCGAACAAGGTGACAGAG	CAGGAGAACCAGGAGAACCAGGAG
Runx2	CCGGTCTCCTTCCAGGAT	GGGAACTGCTGTGGCTTC

## Data Availability

All data included in this study are available upon request by contact with the corresponding author.
